# Molecular characterization of bovine leukemia virus from Moldovan dairy cattle

**DOI:** 10.1007/s00705-017-3241-4

**Published:** 2017-02-17

**Authors:** Aneta Pluta, Marzena Rola-Łuszczak, Piotr Kubiś, Svetlana Balov, Roman Moskalik, Bhudipa Choudhury, Jacek Kuźmak

**Affiliations:** 1 OIE Reference Laboratory for EBL, Department of Biochemistry, National Veterinary Research Institute, Pulawy, Poland; 2 Republican Center for Veterinary Diagnostic, Chisinau, Moldova; 3 Scientific Practical Institute for Biotechnologies and Zootechny and Veterinary Medicine, Chisinau, Moldova; 4 OIE Reference Laboratory for EBL, Department of Virology, Animal and Plant Health Agency, Weybridge, UK

## Abstract

Bovine leukemia virus (BLV) is the causative agent of enzootic bovine leukosis (EBL), a disease that has worldwide distribution. Whilst it has been eradicated in most of Western Europe and Scandinavia, it remains a problem in other regions, particularly Eastern Europe and South America. For this study, in 2013, 24 cattle from three farms in three regions of Moldova were screened by ELISA and nested PCR. Of these cattle, 14 which were PCR positive, and these were molecularly characterized based on the nucleotide sequence of the *env* gene and the deduced amino acid sequence of the encoded gp51 protein. Our results demonstrated a low level of genetic variability (0-2.9%) among BLV field strains from Moldova, in contrast to that observed for other retroviruses, including human immunodeficiency virus (HIV) (20-38%) Mason IL (Trudy vologod moloch Inst 146–164, 1970) and equine infectious anemia virus (EIAV) (~40%) Willems L et al (AIDS Res Hum Retroviruses 16(16):1787–1795, 2000), where the envelope gene exhibits high levels of variation Polat M et al (Retrovirology 13(1):4, 2016). Sequence comparisons and phylogenetic analysis revealed that BLV genotype 7 (G7) is predominant in Moldova and that the BLV population in Moldovan cattle is a mixture of at least three new sub**-**genotypes: G7D, G7E and G4C. Neutrality tests revealed that negative selection was the major force operating upon the 51-kDa BLV envelope surface glycoprotein subunit gp51, although one positively selected site within conformational epitope G was detected in the N-terminal part of gp51. Furthermore, two functional domains, linear epitope B and the zinc-binding domain, were found to have an elevated ratio of nonsynonymous to synonymous codon differences. Together, these data suggest that the evolutionary constraints on epitopes G and B and the zinc-binding domains of gp51 differ from those on the other domains, with a tendency towards formation of homogenous genetic groups, which is a common concept of global BLV diversification during virus transmission that may be associated with genetic drift.

## Introduction

Bovine leukemia virus (BLV), a lymphotropic retrovirus belonging to the genus *Deltaretrovirus* of the family *Retroviridae*, is the etiologic agent of enzootic bovine leucosis (EBL). Infection with BLV may remain clinically silent in an aleukemic form, but about 30% of infected cattle develop persistent lymphocytosis, characterized by polyclonal expansion of B cells, with only a few percent developing lymphoid tumours [1]. BLV infection has a worldwide distribution, and epidemiological studies based on serology show high prevalence in North and South America, some Asiatic and Middle Eastern countries, and Eastern Europe [2–5]. Successful eradication programs have eliminated the disease from most Western European countries, giving these countries a significant trading advantage.

The BLV envelope glycoprotein, encoded by the envelope (*env*) gene, is crucial to the virus life cycle and is the primary target of neutralizing antibodies. Studies of the genetic diversity of the *env* gene of BLV isolates from different geographical locations initially revealed eight different genotypes [2, 6–9]. Recently, Lee *et al.* [10] and Polat *et al.* [11], using whole-genome next-generation sequencing (NGS) analysis, performed a comparative analysis of the *env* sequences and identified two new genotypes, bringing the total to ten (e.g., G1-G10). These authors reported that the homology amongst BLV isolates was 94.5-97.7%.

The envelope glycoprotein consists of a 51-kDa extracellular surface subunit (SU, gp51) and a 30-kDa transmembrane subunit (TM, gp30). Analysis of the nucleotide sequence and the deduced amino acid sequence of the gp51-encoding region identified some distinct and highly conserved regions common to all field isolates of the virus [12, 13]. Some of the mutations found in gp51 were associated with marked changes in the course of infection, and it was postulated that some of them can lead to increased virulence by allowing escape from host immune surveillance [12, 14].

Glycoprotein gp51 contains seven epitopes, named beginning from the N- to the C-terminal end, G, H, F, E-E’, B-B’, D-D’ and A, and three of these (G, H and F) are conformational epitopes that are major determinants of virus neutralization and inhibition of syncytium formation [15]. Additionally, three neutralization domains, ND1, ND2 and ND3, were shown to induce BLV-neutralizing antibodies [16]. The N-terminal and internal regions of gp51 contain five T-cell epitopes that are involved in the induction of proliferative responses in BLV-infected cattle: a CD4^+^ T-cell epitope, a CD8^+^ T-cell epitope, gp51N5, gp51N11 and gp51N12, an immunologic target of cytotoxic T lymphocytes (CTL) [17]. The glycoprotein gp30 contains an extracellular domain encompassing the hydrophobic fusion peptide and a cytoplasmic domain with a YXXL motif that is believed to be involved in signal transduction pathways [18].

We previously showed that while strains from Poland, Russia, Ukraine and Belarus can be assigned mainly to G4 and G7, a few of them contained variations that defined a new genotype designated G8 [8]. Since G8 was a unique genotype that grouped BLV isolates from the Balkan region but no other regions of Eastern Europe, we wanted to determine whether strains from cattle in another Balkan country, Moldova, might also belong to this genotype [6, 8]. EBL was first identified in Moldova in 1965, and subsequent serological surveys confirmed widespread distribution of BLV infection in Moldovan cattle [19, 20]. Moldova launched an eradication program in 2008 that decreased the overall number of BLV-infected cattle from 48.8% to 4.9%. However, serologically positive animals are currently found in the central and western parts of the country, where the regional herd-level prevalence in dairy farms is estimated to be 20% [21].

In this study, we report the genetic variation in BLV strains from this part of the world. We focused on analysis of an 804-base-pair (bp) fragment of the *env* gene including the entire gp51 coding sequence in fourteen BLV samples from Moldovan dairy cattle. The newly generated sequences were analyzed taking into account all currently known functional domains and epitopes of the gp51 surface glycoprotein. Phylogenetic analysis was performed using all currently known BLV genotypes.

## Materials and methods

### Sample collection and DNA isolation

Blood samples were obtained from 24 serologically positive cattle that were naturally infected with BLV, with diagnosis confirmed by an ELISA test (INGEZIM BLV Compac 1.2.BLV.K.3, INGENASA, Spain). The animals came from three herds that were located in three geographically distinct regions of Moldova: Riscani, Aneii Noi, and Hincesti, with 4.4%, 1.0%, and 1.8% cattle seropositivity, respectively. Peripheral blood leukocytes were isolated by centrifugation at 1500 × *g* for 25 minutes, and erythrocytes were haemolysed by osmotic shock with water and 4.5% sodium chloride. After two washes in phosphate-buffered saline (PBS), the supernatant was discarded and the cell pellets were sent to the National Veterinary Research Institute in Pulawy for analysis. Genomic DNA was extracted from 5 × 10^6^ cells using a DNeasy Tissue Kit (QIAGEN) following the manufacturer’s instructions. The concentration of genomic DNA was measured using a nanophotometer (Implen), and the samples were stored at – 20 °C until PCR amplification.

### Amplification of proviral DNA by nested PCR

A fragment of 804 bp of the *env* gene encoding the gp51 surface glycoprotein was amplified by seminested PCR using the following primers: AP_4762 (this study) (5’-GCTCTCCTGGCTACTGACC-3’), ZM2_5786 [22] (5’-TCTGATGGCTAAGGGCAGACACGGC-3’) and ZM5_5733 [22] (5’-GCTAGGCCTAAGGTCAGGGCCGC-3’). Amplification was performed on 1 µg of genomic DNA using KAPA Taq thermal polymerase (KAPA Biosystems) and a thermal cycler (Biometra) with the following cycle parameters: 3 min at 95 °C, 30 s at 95 °C, 30 s at 62 °C (external primers) or 30 s at 66 °C (internal primers), 2 min at 72 °C; after the last (34^th^) cycle, the samples were incubated at 72 °C for 10 min. Reactions were carried out in 50 µl, with 10 µl 10X KAPA Taq Buffer, 1 µl of 10 µM dNTP mix, 1.5 µl of each primer (10 mM), 3 µl of 10 mM MgCl_2,_ and 1.5 U of KAPA Taq DNA Polymerase. Reaction products of all strains were analyzed by 1.5 % agarose gel electrophoresis with ethidium bromide staining (1 µg/ml), in 1X TAE buffer. To rule out the possibility of Taq polymerase errors, we carried out three independent PCR amplifications for each of the DNA templates.

### DNA sequencing and sequence analysis

The PCR products were purified using a NucleoSpin Extract II Kit (Marcherey-Nagel GmbH & Co) and subsequently, three independent PCR products for each sample were sequenced in both directions by the Genomed S A Company (Warsaw, Poland), using a 3730xl DNA Analyzer (Applied Biosystems) and a Big Dye Terminator v3.1 Cycle Sequencing Kit. All raw sequencing data were viewed in BioEdit v7.2.5 (Abbott, Carlsbad C A). The consensus sequences corresponding to the 804-bp fragment of the *env* gene were generated from three repeats of each sample, and they were submitted to the GenBank database under accession numbers KF801457-KF801470 (Table 1). This table also includes 35 BLV reference sequences representing G1-G10, which were used in this study. To estimate the genetic distances among the strains from Moldova (intra-genotype) and between the Moldovan strains and the reference sequences (inter-genotype), analysis based on the maximum composite likelihood model (MEGA 5.2.2) [23, 24] was conducted on the 804-bp sequences. Synonymous and non-synonymous sites were identified using DnaSP 5.0 [25]. Statistical analysis was performed using STATISTICA ver. 10 (StatSoft, Dell Software, USA). A Z-test based on the number of synonymous substitutions per synonymous site (dS) and the number of non-synonymous substitutions per non-synonymous site (dN) was used to test for selection. To assess the relationship between the BLV subtypes identified in this study and those described in previous studies, the 804-bp sequences were aligned to reference sequences using the Geneious Alignment module within Geneious Pro 5.3 Software (Biomatters Ltd) [26], and a phylogenetic tree was constructed by the Bayes method with the GTR substitution model using the tree-builder tool of the Geneious software. For measuring the reliability of the clustering, 1,000 bootstrap replicates were run for each method.

**Table 1:**
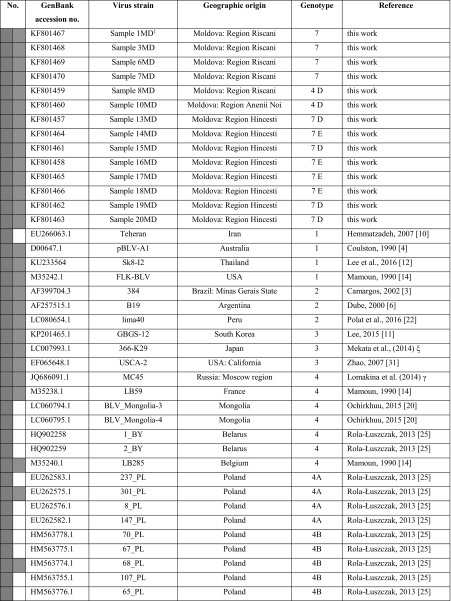

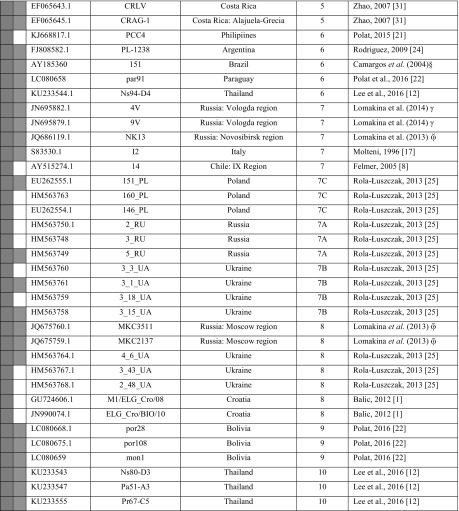
Identification and origin of the sequences used for sequence analysis. The 444-bp and 804-bp sequences are indicated by green and violet squares, respectively (colour figure online)

ξ Mekata et al., 2014,  Lomakina et al., 2013, γ Lomakina et al., 2014, § Camargos et al., 2004, direct submission to GenBank

## Results

### Phylogenetic analysis of the region of BLV *env* encoding gp51

PCR products of the expected size of 804 bp were successfully amplified and sequenced from the proviral DNA of all fourteen strains. Using maximum composite likelihood model analysis, the nucleotide sequences of the fourteen Moldovan strains were aligned together with 35 reference sequences representing BLV genotypes G1-G10. The percentages of nucleotide substitutions were determined by pairwise comparisons (data not shown). All Moldovan sequences were found to be closely related to each other at the nucleotide level, showing an average degree of variability of 1.1%, with a range of variability of 0% to 2.9%. No substantial divergence at the nucleotide level was noted between strains from the same herds and those representing geographically distant herds. Interestingly, the smallest number of base substitutions per site and the shortest genetic distance was observed between Moldovan sequences and those belonging to G4 and G7, with 0.3%-3.6% and 0.2%-3.0% variations, respectively. Based on these results, we inferred that the Moldovan sequences group within G4 and G7.

**Fig. 1:**
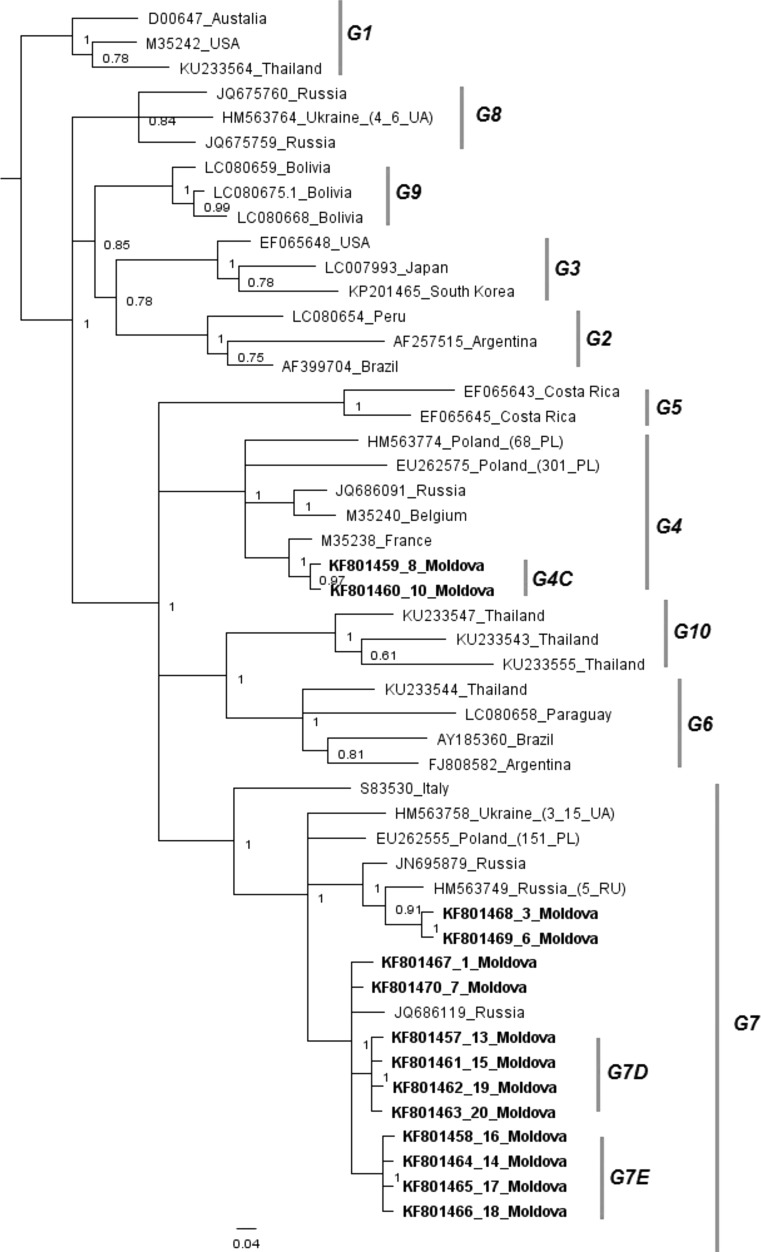
Phylogenetic relationship of the gp51-encoding 804-bp fragment of the *env* gene nucleotide sequences of new BLV subtypes found in this study (bold text) and all known BLV genotypes (n = 49), as inferred by Bayesian analysis. Numbers at nodes indicate posterior probabilities of sampling the node among 11,000 trees. Genotypes and subtypes as well as new subtypes found in this study are indicated at the right by vertical lines

**Fig. 2:**
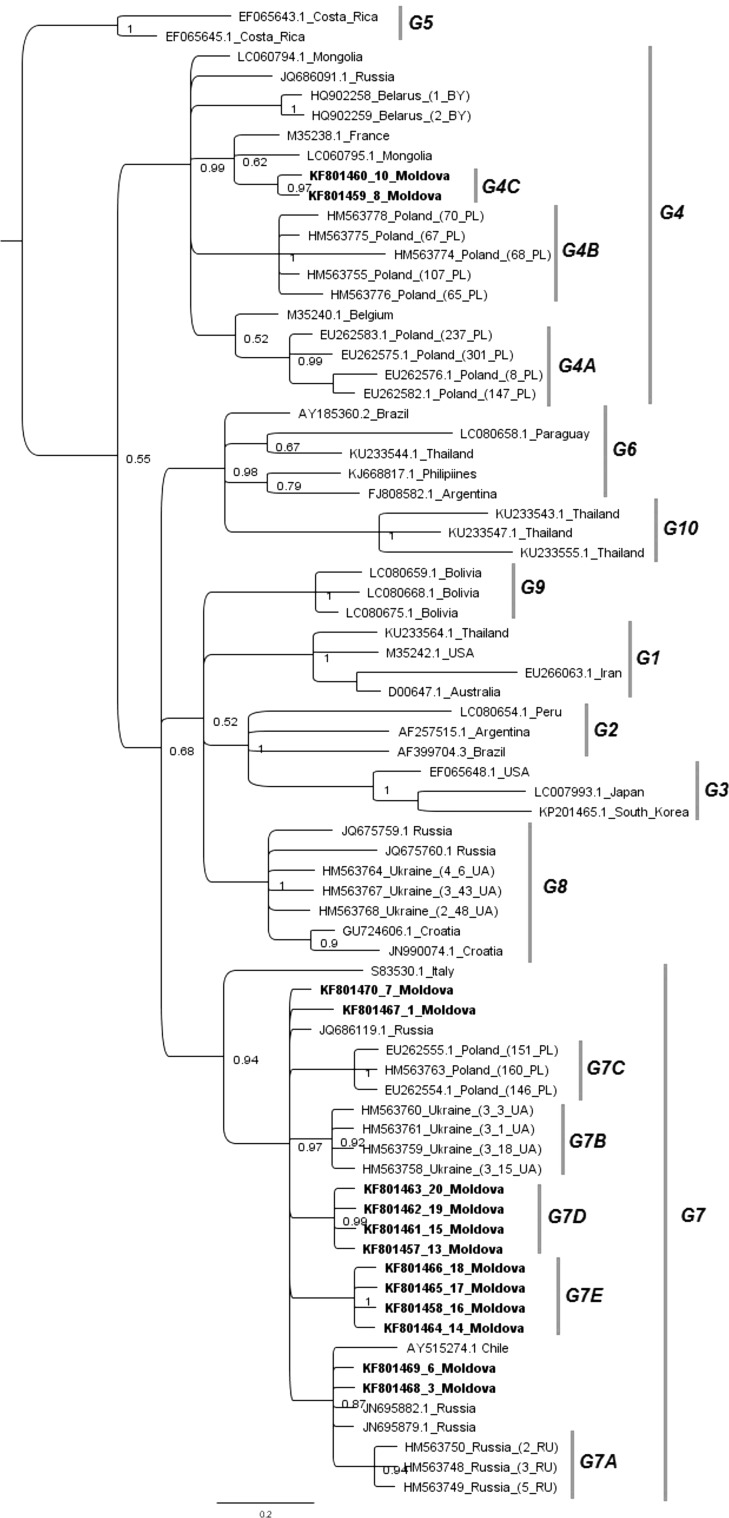
Phylogenetic relationship of a 444-bp fragment of the *env* gene nucleotide sequences of new BLV subtypes found in this study (bold text) and all known BLV genotypes and/or subtypes (n = 75, updated as of August, 2016), as inferred by Bayesian analysis. Numbers at nodes indicate posterior probabilities of sampling the node among 11,000 trees. Subtype terminology is based on that of Rola-Luszczak et al. [8]. Genotypes and/or subtypes and new subtypes found in this study are indicated at the right by vertical lines

To test their affiliation with G4 and G7, phylogenetic trees based on the 804-bp gp51-encoding sequence were constructed using Bayesian methods (Fig. 1). The topology, with high posterior probabilties, indicated that the Moldovan sequences and all reference sequences reported as of August 2016 were clearly classified into ten distinct genotypes (G1 to G10) and confirmed that the fourteen strains from Moldova belong to G4 and G7. For further analysis, we used the tree constructed on 444-bp sub-fragment, since this has been used most often in phylogenetic analysis of BLV and is therefore better for showing the presence of sub-genotypes (Fig. 2).

Twelve of the Moldovan strains grouped together with isolates from Russia, Poland, Chile and Ukraine to form a common branch within G7 with significant posterior probability of 0.94, but they were clearly clustered into distinct groups within this genotype (Fig. 2). Notably, strains 13MD, 15MD, 19MD and 20MD and strains 14MD, 16MD, 17MD and18MD clustered into two clearly distinguished subtypes. Since their existence was supported by high posterior probabilities of 0.99 and 1.00, we propose that the existing topology of G7 be extended to include two additional new subtypes, G7D and G7E, identified in this study and defined by these two clusters of Moldovan strains. Both subtypes co-existed in the same herd from the Hincesti region of central Moldova and showed a maximum divergence of 2.1% in their nucleotide sequences. Another four Moldovan strains, 1MD, 3MD, 6MD and 7MD, from a single herd located in the Riscani region in the northern part of Moldova, also belonged to G7 but did not form well-separated groups and were located close to isolates from Russia and Ukraine. The remaining two strains, 8MD and 10MD, which were from Riscani and Aneii Noi in the southern part of Moldova and were most closely related to isolates from France and Mongolia, were classified together in G4. However, both of these strains formed a separate branch within G4, supported by a significant of posterior probability value 0.97. Therefore, we propose to add sub-genotype G4C to the existing G4A and G4B sub-genotypes. These subtypes showed high posterior probabilities, reflecting variability within G4, with a maximum divergence of 1.6% of nucleotide sequences. Taken together, these results demonstrated that the most prevalent genotype (85.7%) was G7, with the remaining Moldovan strains clustering in G4 (14.3%). Most of the strains formed unique and distinct subtypes correlating with their geographical origin, which we propose to be designated as new sub-genotypes G7D, G7E and G4C.

### Nucleotide and amino acid sequence diversity of the region of BLV *env* encoding gp51

**Fig. 3:**
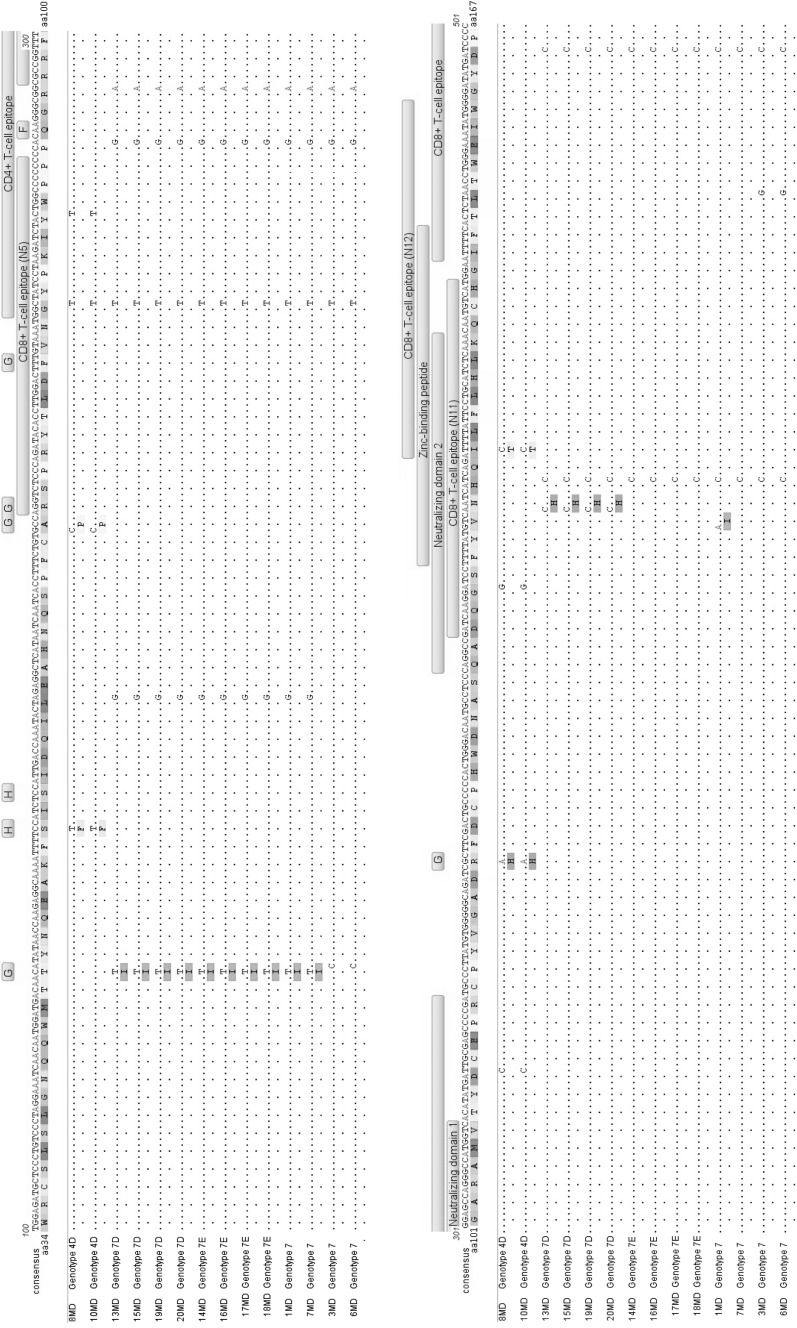

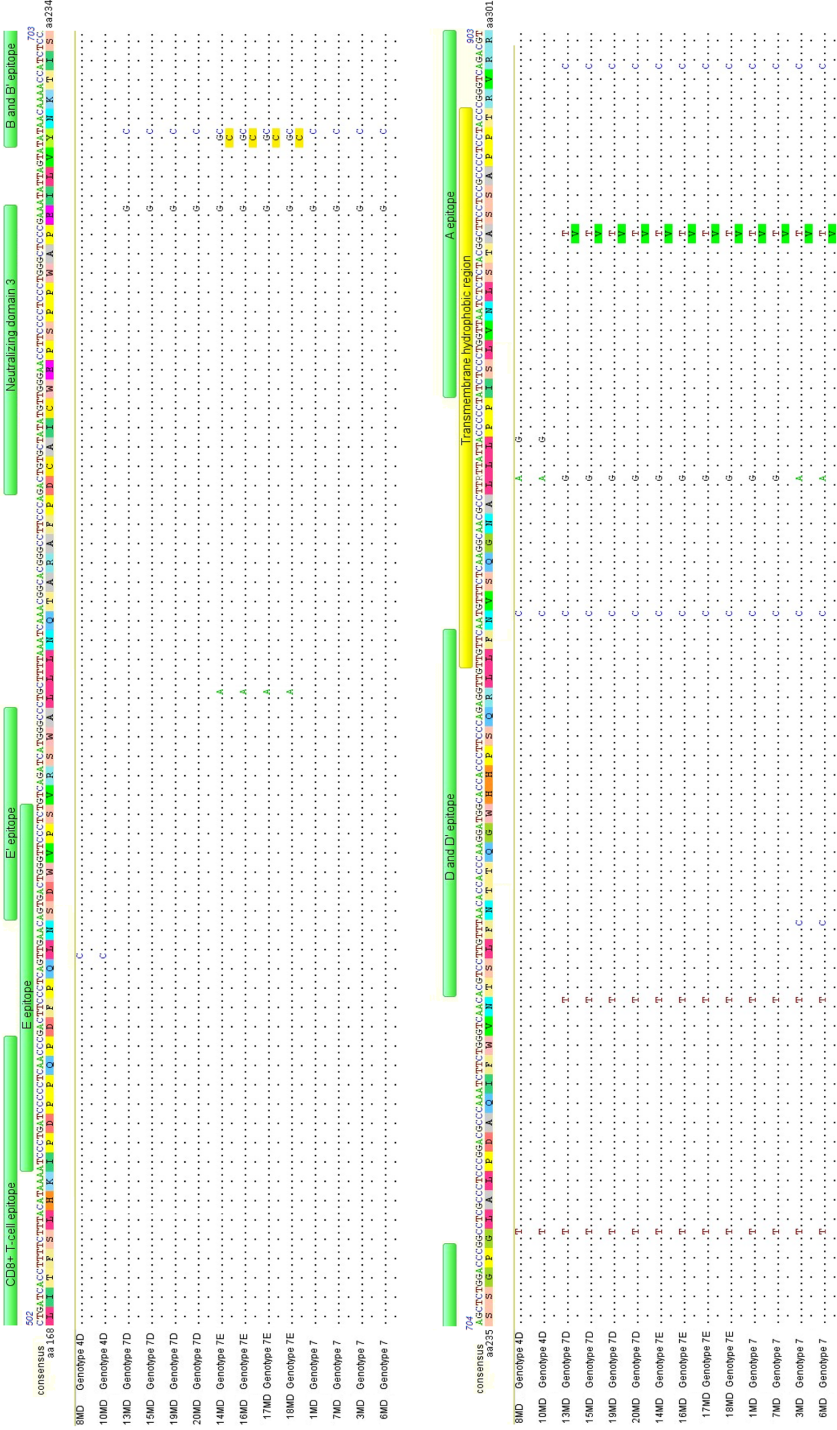
Alignment of the deduced amino acid sequences of glycoprotein gp51 of fourteen Moldovan strains. Differences from the consensus sequence are indicated as is the distribution of corresponding antigenic determinants along the surface glycoprotein gp51. Horizontal bars above the nucleotide sequence alignment indicate the zinc-binding peptide and the antigenic determinants epitopes B, B’, E, E’ (linear), G (conformational), ND1, 2, 3 – neutralization domain, CD4^+^, CD8^+^ T-cell epitopes (green), and the TMHR transmembrane hydrophobic region (yellow) (colour figure online)

The mutations found in BLV strains from Moldovan cattle were mapped in the alignment shown in Fig. 3, with antigenic determinants indicated for comparison and clarity. Comparison to the consensus sequence revealed that 31 out of 804 nucleotide sites (3.9%) had a point variation in at least one Moldovan strain. The nucleotide variability (number of variations per site) in an individual strain varied from 1.6% to 2.1%, with an average value of 1.96%. Since nucleotide sequence variability was found throughout the *env* sequences, we analyzed the significance of these variations with respect to selection pressure on the *env* gene and individual sub-regions of gp51. To perform this analysis, the dN/dS ratio was calculated for each site, and the Z-test was used for selection testing. The results are shown in Table 2. While Z-test results showed extremely high dN/dS (>1) values for neutralizing epitope G, indicating its strong positive selection, negative selection (dN<dS) was found for the gp51-encoding sequence of the *env* gene as a whole and for most of its sub-regions. Although two functional domains, linear epitope B and the zinc-binding domain, showed somewhat higher dN/dS ratios (0.781 and 0.552, respectively), these were not high enough to indicate positive selection. Generally, these results indicated that the regions within *env* encoding functional domains and epitopes of gp51 of Moldovan strains were constrained by strong purifying negative selection.

**Table 2:** DnaSP analysis for evidence of selection in functional domains of BLV gp51 *env* sequences within the Moldovan population

Functional domains		dS	dN	Z-test	dN/dS ratio	dN/dS ratio*
Whole gp51	Overall	0.0304	0.0045	<0.0001	0.154	0.106
Sequential epitopes	A	0.0147	0.0073	<0.0001	0.501	0.462
	B, B’	0.0359	0.0198	<0.001	**0.552**	**0.078**
	D, D’	0.0223	0.0000	<0.0001	0.000	0.184
	E, E’	0.0167	0.0000	<0.0001	0.000	0.052
	F	0.0000	0.0000	>0.999**	0.000	0.000
	G	0.0756	0.0905	<0.001	**1.198**	**2.787**
	H	0.0000	0.0673	<0.0001	N/A***	0.218
Neutralization domains	ND1	0.0000	0.0000	>0.9999	0.000	0.137
	ND2	0.0452	0.0190	<0.0001	0.421	0.298
	ND3	0.0277	0.0000	<0.0001	0.00000	0.007
T-cell epitopes	CD4^+^	0.0456	0.0000	<0.0001	0.00000	0.036
	CD8^+^	0.0273	0.0000	<0.0001	0.00000	0.028
	CD8^+^ (N5)	0.0175	0.0000	<0.0001	0.00000	0.124
	CD8^+^ (N11)	0.0458	0.0177	<0.0001	0.388	0.194
	CD8^+^ (N12)	0.0219	0.0055	<0.0001	0.253	0.064
Zinc-binding peptide	Zbp	0.0239	0.0187	<0.001	**0.781**	**0.217**
Transmembrane hydrophobic region	TMHR	0.0919	0.0000	<0.0001	0.00000	0.152

* dS/dN calculated for ten genetic groups of BLV G1-G10

** Null hypothesis that dS=dN

*** N/A- not analyzed

Next, we analyzed whether nucleotide substitutions affected the amino acid (aa) composition along the full length of mature gp51, encompassing aa positions 34 to 301 and excluding aa 1–33, which comprise the cleaved signal peptide. A comparison of predicted aa sequences with consensus aa sequence revealed the presence of eight aa substitutions (3%), indicated in Fig. 3 by filled rectangles. While aa substitutions were found over the length of gp51, they mainly clustered in the segment associated with antigenic determinants. Some of the substitutions were observed in multiple samples. Interestingly, four out of eight substitutions occurred within the variable conformational epitopes G and H. We found substitutions of serine to phenylalanine at residue 56 within epitope H, alanine to proline at residue 73 within epitope G, arginine to histidine at residue 121 within epitope G, and isoleucine to threonine at residue 144 within the T-cell epitope CD8+N11, which also overlapped with the zinc-binding peptide and neutralizing domain 2. All of these substitutions were specifically linked to the strains 8MD and 10MD, belonging to the new G4D subtype. Four other aa substitutions were found to be characteristic of strains belonging to G7. In four strains (14MD, 16MD, 17MD and 18MD) classified as subtype G7E, a unique substitution of tyrosine to cysteine at residue 229 was observed within epitope B; this substitution was only observed in Moldovan strains. Similarly, in the four strains grouped together in subtype G7D (13MD, 15MD, 19MD and 20MD), a substitution of asparagine to histidine at residue 141 within epitope CD8+N11 was observed. In addition, the twelve Moldovan strains belonging to G7 shared a substitution of alanine to valine at residue 291, within linear epitope A, and ten of these strains also shared a substitution of threonine to isoleucine at residue 48, within the neutralizing epitope G. These two last substitutions are not specific for Moldovan samples, having been previously described for strains from Russia, Italy and Argentina.

## Discussion

In this study, the portion of the *env* gene encoding the gp51 glycoprotein of BLV was isolated from seropositive cattle in Moldova, and their nucleotide and amino acid sequences were analyzed. Genetic distance analysis of 14 sequences representing BLV isolated in Moldova and 35 sequences representing each of the known genotypes (G1-G10) revealed 95.3% to 99.8% sequence identity at the nucleotide level. Thus, our data confirmed the high degree of conservation of the gp51 *env* gene sequence among geographically different strains, as has been reported previously for many BLV isolates [7, 8, 10, 27–30].

The initial phylogenetic analysis was conducted with an 804-bp fragment of the BLV *env* gene. We aimed to include in this analysis as many geographically diverse strains as possible; however, this was limited by the number of available sequences representing the full-length gp51. Therefore, we needed to confirm these results for inferring the phylogenetic tree using a 444-bp fragment of gp51-encoding sequences which has been used most often in phylogenetic analysis of BLV [14, 30–32]. Another advantage of the use of the 444-bp fragment was that we were able to fully match phylogenetic data already generated in our previous study with BLV obtained from Eastern Europe and Siberia [8]. The topology of this phylogenetic tree clearly showed that Moldovan strains represent well-delineated groups within G4 and G7. Surprisingly, none of the strains from Moldova clustered in G8, the genotype that was thought to be specific for this geographical area. Instead, the Moldovan strains grouped primarily with the strains from the neighbouring countries of Ukraine and Russia [8]. Most of the Moldovan strains (85%) were clustered with G7 strains, and we showed the existence of two new well-defined homogenous sub-genotypes, G7D and G7E, within which clustering occurred according to the place of origin, and they co-existed in the same herds. Correlation between genetic affiliation of the viruses and their geographical location has been demonstrated for many retroviruses, including HTLV-1 [33], FIV [34], and FeLV [35]. Similarly, the presence of more than one genotype of BLV in certain geographical areas has been reported by Camargos *et al*. [36], Zhao *et al.* [28] and Polat *et al.* [37]. In our previous work, we also reported the presence of well-separated BLV genogroups composed of isolates of similar geographical origin with a close genetic relationship to each other [8]. It seems that a tendency towards diversification of the virus into homogenous genetic groups is a common concept of global BLV diversification that results from virus transmission, possibly associated with genetic drift. Once dispersed, the virus is then assimilated within herd populations, and gradually, these became homogenous. Confirmation of such a scenario was found in the presence of the single amino acid substitution of tyrosine to cysteine at residue 229 that is unique to the G7E sub-genotype and the asparagine-to-histidine change at residue 141 that is typical of the G7D sub-genotype. Once introduced into small populations, these substitutions became fixed in the proviral genomes. Since the dissemination of BLV infection is associated mainly with introduction of infected cattle into a herd, we speculate that animal trade that took place between countries belonging to CMEA (Council for Mutual Economic Assistance) played a critical role in modulation of the topology of the phylogenetic tree. The role of animal trade in BLV diversification has been emphasized in some studies [30, 32, 38]. Although we demonstrated that most Moldovan strains were of genotype G7, two strains clustered within G4, in close proximity to isolates from France, Mongolia and Belgium. These strains came from two distinct regions in Moldova where dairy cattle have been extensively back-crossed to Holstein-Fresian cattle, which were originally derived from the Dutch Friesian breed [39]. Since G4 represents the isolates that originally came from France and Belgium, this reinforces the concept of an impact of international animal trade on the distribution of particular genotypes and the topology of phylogenetic trees.

The *env* gene of BLV exhibits a relatively high degree of conservation among different strains overall, which is a distinctive feature of viruses belonging to the genus *Deltaretrovirus* [12, 33, 35]. Our analysis revealed only eight aa substitutions in Moldovan strains concentrated exclusively within known epitopes of the gp51 glycoprotein. This is in agreement with previous studies of isolates from other countries showing that most aa substitutions occur within epitopes rather than at random locations in the surface subunit [11, 28, 32]. The high degree of sequence conservation is consistent with the general finding that the *env* gene is subjected to negative selection [28, 32]. However, strong positive selection has been observed for residues in the G and D-D’ epitopes [28]. Similarly, we found a high dN/dS ratio for epitope G, indicating stringent positive selection for this conformational epitope. Three aa substitutions associated with this epitope highlight its role in virus evolution and the possible involvement of epitope G in the evolution of escape mutants. We also found one substitution within a second conformational epitope, H, at position 56, which was previously observed to influence epitope-H-specific antibody recognition [40]. Substitutions within epitopes G and H have been reported previously by several authors [12, 8, 28, 32, 41], but further studies are needed to determine whether these changes can affect the three-dimensional structure of the gp51 glycoprotein and to investigate their possible impact on the effectiveness of diagnostic serological tests. We speculate that the existence of BLV variants with mutations within epitopes G and H may explain the continued recurrence of BLV infection in cattle caused by variants that circumvent the immune response [14, 42]. We also report here two amino acid substitutions in the sequence of a CD8^+^ T-cell (N11) epitope that were identical to those recently found to induce T-cell-dependent cytotoxicity in BLV-infected calves [17]. These mutations were found in aa sequences deduced from nucleotide sequences of seropositive cattle; therefore, they did not seem to affect the diagnostic capacity of serological tests. However, their significance in the context of disease progression and host cellular anti-viral immunity remains to be elucidated. Dube *et al*. [43] described a unique mutation, E161G, in the C-terminal part of a CD8^+^T-cell epitope of an Argentinian isolate that theoretically could alter the stimulation of the anti-CD8^+^ T-cell response. Furthermore, the CD8^+^ T-cell epitope (N11) overlaps with the zinc-binding peptide, which in all deltaretroviruses is indispensable for viral fusion and infectivity *in vivo* [18]. Thus, any aa changes within this region may strongly affect BLV infectivity.

In summary, here, we assessed the phylogenetic clustering of Moldovan BLV strains into three new subgroups of G4 and G7. Similar to what has been described previously for strains from Eastern Europe and Siberia [8], the Moldovan strains showed a tendency to group according to their geographical origin. Molecular characterization of the *env* gene fragment encoding envelope glycoprotein gp51 of these strains showed relatively high nucleotide sequence conservation, as has been reported for other BLV strains [11, 11, 31]. Nevertheless, we found some nucleotide changes in specific regions of the *env* gene and found that these regions were subjected to positive selection. Further studies will be desired to examine the impact of genetic diversity of BLV on disease progression, virus infectivity, and the capacity of diagnostic tests.
